# Significant association between elevated urine albumin-to-creatinine ratio and increased risk of acute coronary syndrome: a retrospective cross-sectional analysis

**DOI:** 10.1080/07853890.2025.2525393

**Published:** 2025-06-29

**Authors:** Yujie Song, Fangying Yan, Yangjie Yu, Junjie Pan, Wei Shen, Huanchun Ni, Jian Li, Xinping Luo, Yong Li, Haiming Shi, Liwen Bao

**Affiliations:** Department of Cardiovascular Disease, Huashan Hospital, Fudan University, Shanghai, China

**Keywords:** Acute coronary syndrome, chronic kidney disease, urine albumin creatinine ratio, coronary artery stenosis severity

## Abstract

**Background:**

Acute coronary syndrome (ACS) is a major cause of mortality worldwide. Chronic kidney disease (CKD) is associated with cardiovascular disease. However, whether CKD increases the risk of ACS, indicating its effect on plaque rupture or erosion, needs to be elucidated.

**Methods:**

This cross-sectional study analyzed the data from patients with coronary artery disease who underwent percutaneous coronary intervention (PCI) between 2016 and 2020. Patients were categorized according to urinary albumin-to-creatinine ratio (UACR) elevation, estimated glomerular filtration rate (eGFR), or Kidney Disease: Improving Global Outcomes (KDIGO) risk classification. Setting chronic coronary syndrome (CCS) as the control, logistic regression was used to evaluate the associations between elevated UACR, eGFR, KDIGO stage, and ACS. Confounding for adjustment included age, sex, hypertension, diabetes, LDL, triglycerides, heart failure, and coronary artery disease-reporting and data system (CADRADS) score.

**Results:**

This cross-sectional study included 1,137 patients with available UACR data (62.9%) from a total of 1806 coronary artery disease (CAD) subjects. Microalbuminuria and macroalbuminuria were associated with an increased risk of ACS (OR = 1.63, 95% CI: 1.15–2.32, *p* = 0.007 and OR = 2.07, 95% CI: 1.18–3.62, *p* = 0.011). Decreased eGFR, elevated UACR, and higher KDIGO stage were correlated with the severity of coronary artery stenosis, and patients with a UACR≥ 300 mg/g had the most severe stenosis (OR, 1.74; 95% CI: 1.07–2.83, *p* = 0.026). Elevated UACR remained correlated with ACS, even after adjusting for the CADRADs score.

**Conclusions:**

Elevated UACR is significantly associated with ACS, suggesting a potential mechanistic role of UACR elevation in plaque rupture or erosion. Early UACR monitoring in CCS is important for preventing ACS.

## Introduction

Coronary artery disease (CAD) is a leading cardiovascular condition worldwide, encompassing both acute coronary syndrome (ACS) and chronic coronary syndrome (CCS). Compared to CCS, ACS is characterized by plaque instability and rupture, leading to significantly higher mortality rates and public health burdens [1]. It is estimated that over 7 million people are diagnosed with ACS each year globally, highlighting the need for improved risk prediction and prevention [2,3].

The risk of CAD and its prognosis are influenced by various factors including hypertension, diabetes, and dyslipidemia. Among these, chronic kidney disease (CKD) is recognized as a key contributor [4]. Even after adjusting for known risk factors, CKD remains an independent risk factor for CAD [5,6]. However, existing research has primarily focused on the long-term cardiovascular outcomes of CKD in populations with or without additional cardiovascular risk factors. Limited data are available to elucidate whether impaired kidney function adversely affects plaque rupture or erosion in patients with severe but stable coronary stenosis.

Patients with CKD are highly represented among those with ACS, making up a substantial proportion (approximately 20–40%) of ACS admissions [7–9]. Impaired kidney function predicted higher in-hospital and long-term mortality of ACS patients [10,11]. Several risk scores like Intermountain Risk Score (IMRS) include the kidney functions to predict prognosis of patients with ACS [12,13]. Kidney disease can be classified into six stages based on the kidney function index such as estimated glomerular filtration rate (eGFR) and urinary albumin-to-creatinine ratio (UACR) [14]. We conducted this cross-sectional study to evaluate the association between elevated UACR, impaired eGFR, and kidney disease: Improving Global Outcomes (KDIGO) classification and the incidence of ACS compared with CCS, with the aim of understanding the underlying effect of UACR elevation on plaque rupture or erosion. Studies have demonstrated that patients with elevated or decreased eGFR are more likely to have double- and triple-vessel CAD [15,16]. Therefore, coronary artery stenosis severity must be considered when evaluating the association between CKD and ACS.

## Methods

### Study population

This retrospective cross-sectional study used the data from patients with CAD who underwent percutaneous coronary intervention (PCI) at our institution between 2016 and 2020. Adult patients with assessed UACR were included in the analysis. Patients were included in the study if they had a UACR and creatinine measurement. The Chronic Kidney Disease Epidemiology Collaboration equation was used to calculate eGFR. Individuals were excluded on the basis of the following criteria: (1) age under 18 years; (2) missing key information, including age, sex, UACR, serum creatinine, or other potential confounding variables; (3) absence of CADRADS (coronary artery disease-Reporting and Data System) score, indicating that coronary CTA was not performed. The study was conducted in line with the Declaration of Helsinki, Demographic and clinical data were sourced from our clinical database and medical records with approval from the Ethics Committee of Huashan Hospital, Fudan University (No. KY2020-050). The need for informed consent was waived by the Ethics Committee of Huashan Hospital, Fudan University because of the retrospective nature of the study.

### Data categorization

#### ACS definition

The diagnosis of ACS was based on clinical, electrocardiography (ECG), and biochemical criteria. In this study, ACS included type I ST-segment elevated myocardial infarction, non-ST-segment elevated myocardial infarction, and unstable angina. Type I myocardial infarction diagnosis was diagnosed based on the 2018 global myocardial infarction definition. In this study, we only enrolled patients with severe coronary stenosis who underwent PCI procedures.

#### AHF and CHF definition

Acute heart failure (AHF) is characterized by a constellation of symptoms and signs over a period of hours to days. It is typically associated with echocardiographic evidence of cardiac dysfunction and elevated levels of BNP or NT-proBNP, and requires urgent medical intervention.

The diagnosis of Chronic heart failure (CHF) was based on the presence of typical symptoms that persisted for more than one month, along with objective evidence of cardiac dysfunction based on natriuretic peptide or echocardiography.

#### CKD classification and KDIGO stages

We used three methods to classify the CKD stages: albuminuria, impaired eGFR, and KDIGO stages. Albuminuria was defined as UACR >30 mg/g, while microalbuminuria and macroalbuminuria were categorized as UACR of 30–300 mg/g and UACR ≥300 mg/g, respectively. The eGFR was calculated using the CKD–EPI equation, incorporating serum creatinine (Scr), age, and sex. Subjects were categorized into CKD stages: Stage 1 (>90 mL/min/1.73 m^2^), Stage 2 (60–90 mL/min/1.73 m^2^), Stage 3 (30–60 mL/min/1.73 m^2^), Stage 4 (15–30 mL/min/1.73 m^2^), and Stage 5 (<15 mL/min/1.73 m^2^). KDIGO classifies CKD into stages based on eGFR and albuminuria. The KDIGO stages were classified based on eGFR and UACR. Low risk was defined as eGFR ≥60 mL/min/1.73 m^2^ and UACR <30 mg/g. Moderate risk was defined as eGFR ≥60 mL/min/1.73 m^2^ and UACR between 30 and 300 mg/g, or eGFR between 45 and 60 mL/min/1.73 m^2^ and UACR <30 mg/g. High risk was defined as eGFR ≥60 mL/min/1.73 m^2^ and UACR ≥300 mg/g, or eGFR between 45 and 60 mL/min/1.73 m^2^ and UACR between 30 and 300 mg/g, or eGFR between 30 and 45 mL/min/1.73 m^2^ and UACR <30 mg/g. Very high risk was defined as eGFR <60 mL/min/1.73 m^2^ and UACR ≥300 mg/g, or eGFR <45 mL/min/1.73 m^2^ and UACR between 30 and 300 mg/g, or eGFR <30 mL/min/1.73 m^2^ (Supplemental Table 1).

#### Hypertension, diabetes and CADRADs score

Hypertension was categorized based on systolic blood pressure ≥140 mmHg, diastolic blood pressure ≥90 mmHg, or use of antihypertensive medication. Diabetes was defined as a history of diabetes, HbA1c level ≥6.5%, or fasting plasma glucose ≥7.0 mmol/L. The severity of coronary artery stenosis was assessed using the CADRADS score. CADRADS is a standardized system for CAD based on coronary computed tomography angiography (CTA). The grades are as follows: CADRADS 0: No CAD; CADRADS 1: Non-obstructive CAD with minimal plaque and less than 50% stenosis; CADRADS 2: Mild CAD with 50–69% stenosis; CADRADS 3: Moderate CAD with 70–89% stenosis; CADRADS 4: Severe CAD with 90% or greater stenosis; CADRADS 5: Complete or near-total occlusion of a coronary artery.

### Statistical analysis

Continuous variables are presented as medians with interquartile ranges, while categorical variables are expressed as numbers (percentages). Data distribution was assessed using the Shapiro–Wilk test. Comparisons between groups were conducted using unpaired two-tailed *t* tests for normally distributed variables or Mann–Whitney test for non-normally distributed variables. One-way ANOVA was used for comparisons across multiple groups, and categorical variables were analyzed using the chi-squared test. Logistic regression models were used to assess the associations between eGFR, UACR, KDIGO stages, and ACS, adjusting for confounding factors such as age, sex, hypertension, diabetes, LDL, and triglycerides. The CADRADS score was included as a covariate in further analyses. Statistical significance was defined as a two-sided *P* value <0.05. Statistical analyses were performed using Stata (version 18.0), R (version 4.1.3), and GraphPad Prism (version 9.5.0) software.

## Results

### Baseline characteristics

In total, 1,137 subjects (62.9%) with screened UACR, derived from 1,806 CAD subjects who underwent PCI, were included in the final analysis. The median age was 66.0 years (IQR, 58.0–73.0), comprising 74.7% male. The median UACR was 9.97 mg/g (IQR, 5.40–29.2 mg/g). Medical complications included hypertension and diabetes in 74.1% and 44.2% of the subjects, respectively. ACS was present in 26.3% of participants, along with additional cases of acute heart failure (1.23%) and chronic heart failure (7.66%). The prevalence of stroke has not yet been directly reported (Table 1).

**Table 1.** Demographics and clinical characteristics of patients according to albuminuria severity.

	Total	UACR < 30	30 < UACR < 300	UACR > 300	*p* value
	*n* = 1,137	*n* = 860	*n* = 208	*n* = 69	
Male	849 (74.7%)	656 (76.3%)	145 (69.7%)	48 (69.6%)	0.089
Age	66.0 [58.0; 73.0]	65.0 [58.0; 72.0]	68.0 [60.0; 77.0]	67.0 [60.0; 74.0]	0.006
UACR	9.97 [5.40; 29.2]	7.13 [4.83; 11.9]	61.1 [41.8; 106]	977 [476; 1874]	<0.001
**Medical history**					
Hypertension	842 (74.1%)	605 (70.3%)	177 (85.1%)	60 (87.0%)	<0.001
Diabetes	502 (44.2%)	341 (39.7%)	106 (51.0%)	55 (79.7%)	<0.001
Acute coronary syndrome	299 (26.3%)	207 (24.1%)	67 (32.2%)	25 (36.2%)	0.009
Acute heart failure	14 (1.23%)	5 (0.58%)	6 (2.90%)	3 (4.35%)	0.002
Chronic heart failure	87 (7.66%)	50 (5.81%)	23 (11.1%)	14 (20.3%)	<0.001
CADRADS score					0.015
4A	700 (61.6%)	545 (63.4%)	123 (59.1%)	32 (46.4%)	
4B	163 (14.3%)	126 (14.7%)	25 (12.0%)	12 (17.4%)	
5	274 (24.1%)	189 (22.0%)	60 (28.8%)	25 (36.2%)	
CKD stage					
1	625 (55.0%)	530 (61.6%)	83 (39.9%)	12 (17.4%)	
2	386 (33.9%)	290 (33.7%)	77 (37.0%)	19 (27.5%)	
3	100 (8.80%)	39 (4.53%)	41 (19.7%)	20 (29.0%)	
4	18 (1.58%)	1 (0.12%)	6 (2.88%)	11 (15.9%)	
5	8 (0.70%)	0 (0.00%)	1 (0.48%)	7 (10.1%)	
KDIGO stage					
Low risk	820 (72.1%)	820 (95.3%)	0 (0.00%)	0 (0.00%)	
Moderate risk	191 (16.8%)	31 (3.60%)	160 (76.9%)	0 (0.00%)	
High risk	65 (5.72%)	8 (0.93%)	26 (12.5%)	31 (44.9%)	
Very high risk	61 (5.36%)	1 (0.12%)	22 (10.6%)	38 (55.1%)	
**Biochemical results**					
Fasting glucose (mmol/L)	5.80 [5.18; 7.30]	5.70 [5.10; 6.90]	6.30 [5.30; 8.00]	7.29 [5.50; 9.72]	<0.001
HBA1C (%)	6.20 [5.80; 7.10]	6.10 [5.80; 6.80]	6.50 [5.90; 7.50]	7.50 [6.20; 9.17]	<0.001
Triglyceride (mmol/L)	1.46 [1.04; 2.08]	1.41 [1.01; 2.03]	1.56 [1.13; 2.15]	1.78 [1.06; 2.85]	0.007
Cholesterol (mmol/L)	3.88 [3.23; 4.60]	3.83 [3.17; 4.51]	4.15 [3.30; 4.86]	4.13 [3.63; 4.96]	0.003
LDL (mmol/L)	2.28 [1.68; 2.92]	2.24 [1.66; 2.84]	2.37 [1.73; 2.95]	2.56 [1.86; 3.15]	0.039
HDL (mmol/L)	0.97 [0.83; 1.13]	0.99 [0.84; 1.15]	0.93 [0.79; 1.06]	0.93 [0.75; 1.07]	<0.001
Albumin (g/L)	41.0 [38.0; 43.0]	41.0 [39.0; 43.0]	41.0 [37.0; 43.0]	38.0 [34.0; 40.0]	<0.001
**Medications**					
Statins	972 (85.5%)	748 (87.0%)	170 (81.7%)	54 (78.3%)	0.033
Antiplatelet drugs	1034 (90.9%)	792 (92.1%)	182 (87.5%)	60 (87.0%)	0.058
ACEI/ARB	696 (61.2%)	525 (61.0%)	135 (64.9%)	36 (52.2%)	0.167
CCB	219 (19.3%)	153 (17.8%)	40 (19.2%)	26 (37.7%)	<0.001
β blockers	693 (60.9%)	518 (60.2%)	131 (63.0%)	44 (63.8%)	0.678
Anticoagulant	25 (2.20%)	17 (1.98%)	7 (3.37%)	1 (1.45%)	0.457

Kidney function assessment revealed that 11.08% of the patients had impaired eGFR, with 8.80% in stage 3, 1.58% in stage 4, and 0.70% in stage 5. The proportion of patients with normal UACR (<30 mg/g) was 75.6%, 18.3% had microalbuminuria (30–300 mg/g), and 6.1% had macroalbuminuria (>300 mg/g). According to the KDIGO stages, patients with normal UACR were classified as low risk (95.3%), while the microalbuminuria group had 76.9%, 12.5%, and 10.6%, respectively. In the macroalbuminuria group, 44.9% were high risk and 55.1% were at very high risk.

CADRADS scores for coronary artery stenosis showed that 61.6% had a score of 4 A, 14.3% a score of 4B, and 24.1% a score of 5. Among the participants, 85.5% used statins, 90.9% received antiplatelet therapy, and 61.2% used ACE inhibitors or ARBs. None of the patients used sodium–glucose transporter 2 (SGLT2) inhibitors.

Albuminuria is more prevalent among patients with advanced age, hypertension, diabetes, ACS, AHF, and CHF. These individuals exhibit higher levels of fasting glucose, hemoglobin A1c, triglycerides, and LDL, as well as lower levels of HDL and serum albumin. The use of statin therapy was slightly less frequent, whereas calcium channel blocker use was significantly more common in patients with increased UACR. No significant differences were observed in the use of antiplatelet agents, ACEI/ARBs, β-blockers, or anticoagulants between the groups.

**Figure 1.**
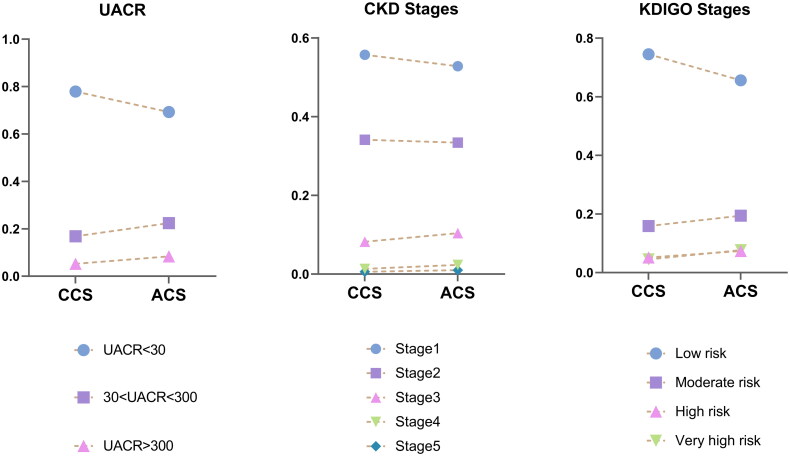
Comparison of UACR, CKD stages, and KDIGO risk categories between ACS and CCS groups. Chi-square test results indicate significant group differences in UACR (**p < 0.01) and KDIGO risk (*p < 0.05), with ACS patients exhibiting higher albuminuria levels and elevated KDIGO risk. No significant difference is observed for CKD stages between ACS and CCS groups.

From another perspective, compared with CCS, ACS had a higher prevalent of elevated UACR (*p* < 0.01) and severe KDIGO stages (*p* < 0.05), whereas no significant differences were observed in CKD stages (Figure 1).

#### The association between elevated UACR, impaired eGFR, KDIGO stage and ACS

Setting CCS as the control, we conducted logistic regression analysis to evaluate the association between elevated UACR, impaired eGFR, KDIGO stage, and ACS compared with subjects with normal kidney function.

**Figure 2.**
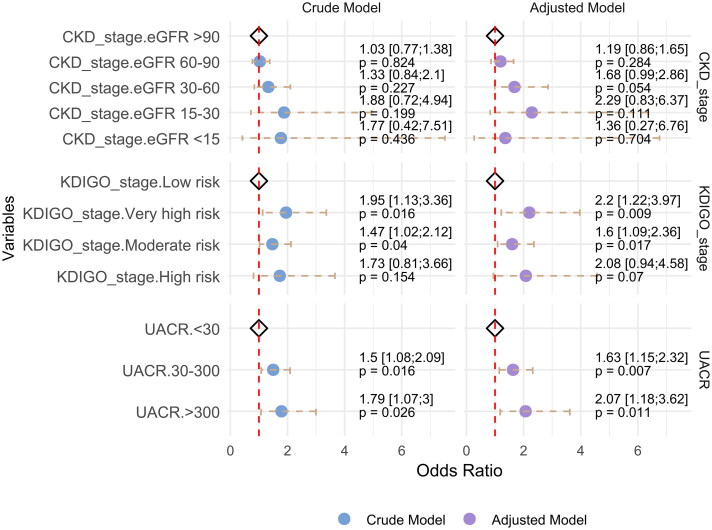
Forest Plot illustrating the odds ratios and 95% CI for the association between UACR, eGFR, and KDIGO stages with the outcome of ACS in crude and adjusted models. The adjusted model accounts for gender, age, hypertension, diabetes, LDL, and TG levels. Each variable within UACR, CKD stage, and KDIGO stage is represented as a point estimate with error bars for the 95% CI. The reference category for each group is shown with a diamond symbol and set at an OR of 1. The red dashed vertical line indicates the null effect (or = 1). The crude Model is represented in blue, while the adjusted Model is in purple.

In univariate analysis, microalbuminuria (OR: 1.50, 95% CI: 1.08–2.09, *p* = 0.016) and macroalbuminuria (OR: 1.79, 95% CI: 1.07–3.00, *p* = 0.026) were associated with an increased risk of ACS (Figure 2). After adjusting for age and sex, albuminuria remained significantly associated with increased ACS risk (microalbuminuria vs. non-albuminuria, OR: 1.58; 95% CI: 1.13–2.21, *p* = 0.008; macroalbuminuria vs. non-albuminuria, OR: 1.89, 95% CI: 1.13–3.19, *p* = 0.016) (Supplemental Table 2). After further adjustment for age, sex, diabetes, hypertension, LDL, triglycerides, and heart failure, the odds ratios were 1.63 for microalbuminuria (95% CI: 1.15–2.32, *p* = 0.007) and 2.07 for macroalbuminuria (95% CI: 1.18–3.62, *p* = 0.011) were calculated (Figure 2).

For eGFR, an elevated risk of ACS was observed from CKD stage 2 to CKD stage 4 compared to CKD stage 1 after adjusting for age, sex, diabetes, hypertension, LDL, triglycerides, and heart failure. The odds ratios was 1.19 (95% CI: 0.86–1.65, *p* = 0.284) for CKD stage 2, 1.68 (95% CI: 0.99–2.86, *p* = 0.054) for CKD stage 3, and 2.29 (95% CI: 0.83–6.37, *p* = 0.111) for CKD stage 4, although these associations did not reach statistical significance. No clear trend was observed for CKD stage 5 (OR: 1.36, 95% CI: 0.27–6.76, *p* = 0.704), potentially due to the limited sample size in this group.

After adjusting for confounding variables, the risk of ACS was significantly elevated in the moderate-risk group (OR: 1.56, 95% CI: 1.08–2.26, *p* = 0.018) than in the low-risk group. This risk further increased in the high-risk group (OR: 2.01, 95% CI: 1.12–3.62, *p* = 0.019) and very high-risk group (OR: 2.27, 95% CI: 1.25–4.12, *p* = 0.007).

#### Elevated UACR, impaired eGFR, KDIGO stage are indicators for coronary artery stenosis severity

**Figure 3.**
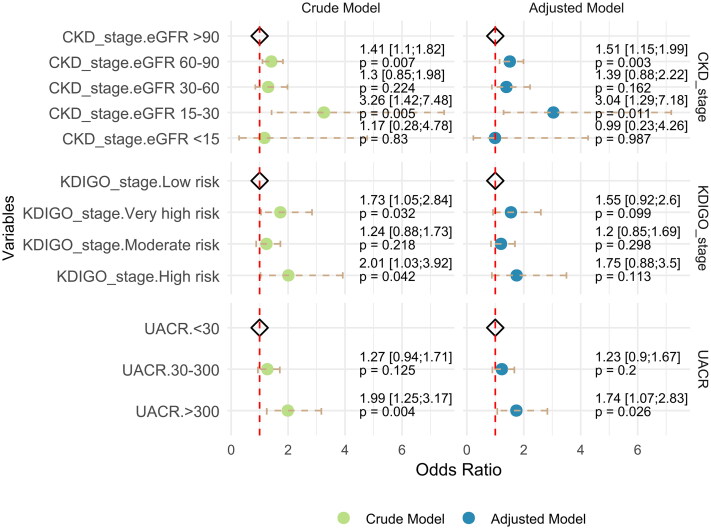
Forest plot illustrating the odds ratios and 95% CI for the association between UACR, eGFR, and KDIGO stages with the coronary artery stenosis severity (CADRADS score) in crude and adjusted models. The adjusted model accounts for gender, age, hypertension, diabetes, LDL, and TG levels. The diamond symbols represent reference categories, set at OR = 1, indicated by the red dashed vertical line. The crude model is represented in green, while the adjusted model is in blue.

In the regression analysis, an elevated UACR was associated with coronary artery stenosis (Figure 3; Supplemental Table 3). Patients with microalbuminuria (30–300 mg/g) had a 1.27-fold increased likelihood of severe CAD compared to those with normal UACR (<30 mg/g) (OR: 1.27; 95% CI: 0.94–1.71, *p* = 0.125). The risk was higher and reach statistical significance for macroalbuminuria (>300 mg/g), with an odds ratio of 1.99 (95% CI: 1.25–3.17, *p* = 0.004). After adjusting for age, gender, hypertension, diabetes, TG, LDL, and heart failure, the adjusted odds ratio was 1.23 (95% CI: 0.90–1.67, *p* = 0.200) for microalbuminuria and 1.74 (95% CI: 1.07–2.83, *p* = 0.026) for macroalbuminuria.

Decreased eGFR was also associated with coronary artery stenosis. Compared to patients with CKD stage 1, those with CKD stage 2 had a higher risk of severe coronary artery stenosis (OR: 1.51; 95% CI: 1.15–1.99, *p* = 0.003). CKD stage 4 was associated with a significantly higher risk of coronary artery stenosis (OR: 3.04; 95% CI: 1.29–7.18, *p* = 0.011), while no significant association was found in CKD stage 5 (*p* = 0.987).

For the KDIGO stages, the risk of ACS showed a progressive increase from moderate to very high risk compared with the low-risk group after adjustment. The odds ratios were 1.19 (95% CI: 0.86–1.64, *p* = 0.301) for moderate risk, 1.40 (95% CI: 0.84–2.33, *p* = 0.200) for high risk, and 1.64 (95% CI: 0.98–2.76, *p* = 0.061) for very high risk. While this trend suggested an increasing risk of ACS with worsening KDIGO stage, statistical significance was observed only in the unadjusted model for the very high-risk group (*p* = 0.019).

#### Strong association between elevated UACR and ACS independent of coronary artery stenosis severity

**Figure 4.**
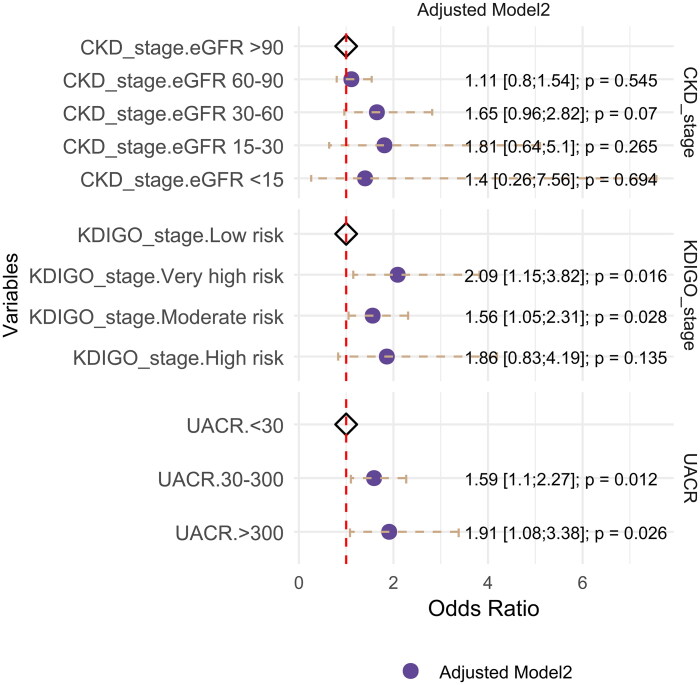
Forest plot illustrating the odds ratios and 95% CI for the association between UACR, eGFR, and KDIGO stages with ACS in the model adjusted for gender, age, hypertension, diabetes, LDL, TG, and CADRADS score. The crude model is represented in purple.

After integrating the CADRADS score as an indicator of coronary artery stenosis severity into the logistic regression model, we reassessed the association between the UACR, eGFR, KDIGO stage, and ACS (Figure 4; Supplemental Table 4). This association was attenuated after adjustment for the CADRADS score. Nonetheless, increased UACR remained associated with an increased risk of ACS, exhibiting an odds ratio of 1.59 for microalbuminuria (95% CI: 1.10–2.27, *p* = 0.012) and 1.91 for macroalbuminuria (OR: 1.91, 95% CI: 1.08–3.38, *p* = 0.026).

For eGFR, no statistically significant associations with ACS risk were observed across the different categories compared with the reference group after adjustment. The odds ratios were 1.11 (95% CI: 0.80–1.54, *p* = 0.545) for CKD stage 2, 1.65 (95% CI: 0.96–2.82, *p* = 0.070) for CKD stage 3, 1.81 (95% CI: 0.64–5.10, *p* = 0.265) for CKD stage 4, and 1.40 (95% CI: 0.26–7.56, *p* = 0.694) for CKD stage 5.

For the KDIGO stages, a progressive increase in ACS risk was observed. Compared to the low-risk group, the adjusted odds ratios were 1.52 (95% CI: 1.05–2.22, *p* = 0.028) for moderate risk, 1.91 (95% CI: 1.05–3.49, *p* = 0.035) for high risk, and 2.14 (95% CI: 1.17–3.92, *p* = 0.014) for very high risk, indicating significant associations.

## Discussion

This study examined the association between CKD, especially elevated UACR, and an increased risk of ACS, indicating the potential mechanism of elevated UACR on plaque rupture or erosion. The novel aspects of this study are as follows. First, while most studies have focused on the association between elevated UACR and coronary artery stenosis severity or CAD prognosis, our cross-sectional analysis compared ACS risk with CCS in patients undergoing PCI to evaluate the effect on plaque rupture or erosion. Second, we evaluated the association between ACS risk and CKD from three perspectives: the UACR, eGFR, and KDIGO classification. Our findings indicated that microalbuminuria and macroalbuminuria are associated with an increased risk of ACS. Although no statistically significant association was found between decreased eGFR and ACS risk, a trend of elevated risk was observed. Previous research reported that CKD stages 2 and above were associated with a higher risk of myocardial infarction [17]. Our findings suggest that early UACR monitoring should also be recommended in the CAD population.

Increased UACR is recognized as a risk factor for cardiovascular disease in the general population, but it is also linked to conventional risk factors, such as age, sex, hypertension, and diabetes. Therefore, it is essential to evaluate albuminuria while controlling for these factors. Some studies have shown that UACR remains a cardiovascular risk factor in the general population without diabetes or hypertension [5,6]. Our findings align with this finding, demonstrating that albuminuria significantly increased ACS risk compared to CCS, even after adjusting for hypertension and diabetes. Albuminuria is also associated with the severity of coronary artery stenosis [15,18]. A longitudinal study found that albuminuria contributes to increased vascular events after adjusting for baseline atherosclerosis, distinguishing between stenosis of ≥50% or <50% [19]. Our study, using the CADRADS score, showed similar results, indicating that albuminuria is associated with a higher incidence of ACS even after adjusting for stenosis severity.

Cardiologists commonly use the eGFR to assess renal function in patients with CVD. However, our data indicate that UACR should also be considered for further risk assessment of ACS, particularly in CAD and early renal function impairment. Recent studies have shown that in addition to ACEI/ARB, patients with elevated UACR can benefit from SGLT2 inhibitors and finerenone in protecting cardiac function. Trials such as DAPA–CKD and EMPA–Kidney have demonstrated that by lowering the elevated UACR, SGLT2 inhibitors protect CKD patients from worsening renal function and decompensated heart failure, regardless of their diabetes status [20,21]. Similarly, the FIDELIO–DKD and FIGARO–DKD trials showed significant renal and cardiac function protection with finerenone in diabetics with elevated UACR [22]. However, further research is needed to determine whether CAD patients can benefit from SGLT2 inhibitors and finerenone for the prevention of ACS.

The underlying mechanisms of this association may involve endothelial dysfunction. CKD is associated with multiple processes such as inflammation, oxidative stress and circulating uremic toxins which damage endothelium and cause endothelial dysfunction [23]. Endothelial dysfunction may trigger endothelial apoptosis and plaque erosion, which trigger with approximately 40% of myocardial infarction [24,25]. Many researches have hypothesized that albuminuria reflects widespread endothelial dysfunction, but additional work is needed to uncover whether albuminuria is directly pathologic or causative of cardiovascular disease [26]. The current treatment such as RAAS inhibitors, SGLT-2 inhibitors and GLP-1 receptor agonists all influence pathways involved in endothelial dysfunction [27–29].

Our study has several limitations. First, as a cross-sectional study, we were unable to evaluate the prediction of elevated UACR on the incidence of ACS. Second, the small number of patients with CKD stages 4 and 5 may have affected the association evaluation. Third, UACR data were available for only 62.9% of patients, which may introduce selection bias.

## Conclusion

**Figure 5.**
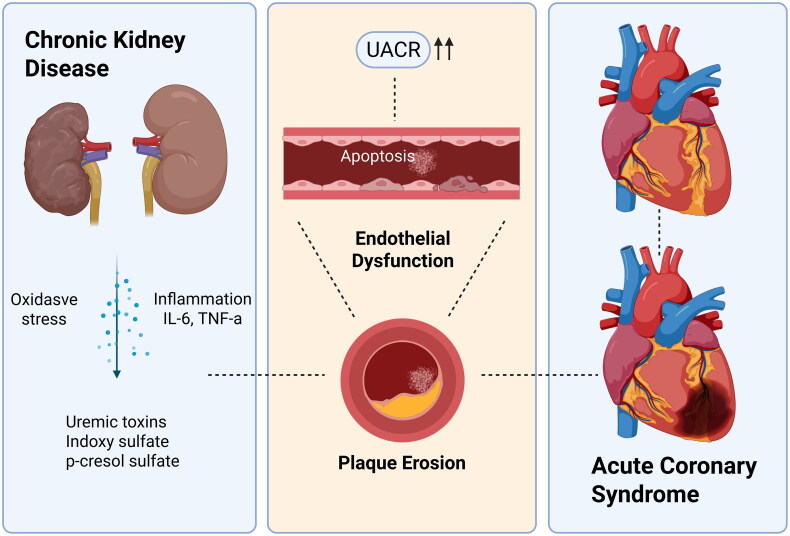
This figure demonstrates the association between elevated UACR and an increased risk of ACS, potentially mediated by oxidative stress- and inflammation-induced endothelial dysfunction. Further studies are needed to establish a causal relationship.

Elevated UACR may serve as a critical indicator of ACS risk compared with CCS risk, even after adjusting for coronary artery stenosis severity. Early UACR monitoring, especially in CKD stages 1 and 2, is vital for identifying high-risk individuals for ACS. Further basic research and prospective cohort studies are warranted to investigate the potential mechanisms by which elevated UACR may contribute to plaque instability in CAD (Figure 5).

## Supplementary Material

Supplemental Material.docx

## Data Availability

Data are available on reasonable request from Liwen Bao (blw_betty@163.com).
