# Supplementary material for: Significant association between elevated urine albumin-to-creatinine ratio and increased risk of acute coronary syndrome: a retrospective cross-sectional analysis
Source: Ann Med. 2025 Jun 29;57(1):2525393. doi: 10.1080/07853890.2025.2525393 (PMC12207781; doi:10.1080/07853890.2025.2525393)
Supplement: Supplemental Material.docx [file IANN_A_2525393_SM3276.docx]

**Supplementary Materials**

**Supplemental table 1. KDIGO Risk Stratification Matrix Based on eGFR and UACR.**

| **eGFR**  **(ml/min/1.73m2)/ UACR (mg/g)** | **A1 (<30)** | **A2 (30–300)** | **A3 (>300)** |
| --- | --- | --- | --- |
| G1 (≥90) | Low risk | Moderate risk | High risk |
| G2 (60–89) | Low risk | Moderate risk | High risk |
| G3a (45–59) | Moderate risk | High risk | Very high risk |
| G3b (30–44) | High risk | Very high risk | Very high risk |
| G4 (15–29) | Very high risk | Very high risk | Very high risk |
| G5 (<15) | Very high risk | Very high risk | Very high risk |

**Supplemental table 2. Logistic regression models for the association between UACR, eGFR, KDIGO stage and ACS incidence.**

|  | **Model 1(a)** | **p value** | **Model 1(b)** | **p value** | **Model 1(c)** | **p value** |
| --- | --- | --- | --- | --- | --- | --- |
| **UACR (mg/g)** |  |  |  |  |  |  |
| <30 | reference |  | reference |  | reference |  |
| 30-300 | 1.50 [1.08;2.09] | 0.016 | 1.58 [1.13;2.21] | 0.008 | 1.63 [1.15;2.32] | 0.007 |
| >300 | 1.79 [1.07;3.00] | 0.026 | 1.89 [1.13;3.19] | 0.016 | 2.07 [1.18;3.62] | 0.011 |
| **eGFR**  **(ml/min/1.73m2)** |  |  |  |  |  |  |
| >90 | reference |  | reference |  | reference |  |
| 60-90 | 1.03 [0.77;1.38] | 0.824 | 1.15 [0.84;1.56] | 0.385 | 1.19 [0.86;1.65] | 0.284 |
| 30-60 | 1.33 [0.84;2.10] | 0.227 | 1.56 [0.95;2.55] | 0.076 | 1.68 [0.99;2.86] | 0.054 |
| 15-30 | 1.88 [0.72;4.94] | 0.199 | 2.11 [0.79;5.62] | 0.137 | 2.29 [0.83;6.37] | 0.111 |
| <15 | 1.77 [0.42;7.51] | 0.436 | 1.71 [0.40;7.29] | 0.466 | 1.36 [0.27;6.76] | 0.704 |
| **KDIGO stage** |  |  |  |  |  |  |
| Low risk | reference |  | reference |  | reference |  |
| Moderate risk | 1.39 [0.98;1.97] | 0.064 | 1.46 [1.03;2.07] | 0.036 | 1.56 [1.08;2.26] | 0.018 |
| High risk | 1.63 [0.95;2.97] | 0.076 | 1.87 [1.08;3.25] | 0.025 | 2.01 [1.12;3.62] | 0.019 |
| Very high risk | 1.93 [1.12;3.31] | 0.018 | 2.08 [1.20;3.61] | 0.009 | 2.27 [1.25;4.12] | 0.007 |

a Crude Model

b Adjusted for age and gender.

c Adjusted for gender, age, hypertension, diabetics, LDL, TG, CHF, and AHF.

**Supplemental table 3. Logistic regression models for the association between UACR, eGFR, KDIGO stage and CADRADS score.**

|  | **Model 2(a)** | ***p* value** | **Model 2(b)** | ***p* value** |
| --- | --- | --- | --- | --- |
| **UACR (mg/g)** |  |  |  |  |
| <30 | reference |  | reference |  |
| 30-300 | 1.27 [0.94;1.71] | 0.125 | 1.23 [0.90;1.67] | 0.200 |
| >300 | 1.99 [1.25;3.17] | 0.004 | 1.74 [1.07;2.83] | 0.026 |
| **eGFR**  **(ml/min/1.73m2)** |  |  |  |  |
| >90 | reference |  | reference |  |
| 60-90 | 1.41 [1.10;1.82] | 0.007 | 1.51 [1.15;1.99] | 0.003 |
| 30-60 | 1.30 [0.85;1.98] | 0.224 | 1.39 [0.88;2.22] | 0.162 |
| 15-30 | 3.26 [1.42;7.48] | 0.005 | 3.04 [1.29;7.18] | 0.011 |
| <15 | 1.17 [0.28;4.78] | 0.830 | 0.99 [0.23;4.26] | 0.987 |
| **KDIGO stage** |  |  |  |  |
| Low risk | reference |  | reference |  |
| Moderate risk | 1.22 [0.89;1.67] | 0.221 | 1.19 [0.86;1.64] | 0.301 |
| High risk | 1.47 [0.91;2.40] | 0.118 | 1.40 [0.84;2.33] | 0.200 |
| Very high risk | 1.81 [1.10;2.97] | 0.019 | 1.64 [0.98;2.76] | 0.061 |

a Crude model.

b Adjusted for gender, age, hypertension, diabetics, LDL, TG, CHF, and AHF.

**Supplemental table 4. Logistic regression models for the association between UACR, eGFR, KDIGO stage and ACS incidence after adjusting for the CADRADs score.**

|  | **Model 1(d)** | ***p* value** |
| --- | --- | --- |
| **UACR (mg/g)** |  |  |
| <30 | reference |  |
| 30-300 | 1.59 [1.10;2.27] | 0.012 |
| >300 | 1.91 [1.08;3.38] | 0.026 |
| **eGFR**  **(ml/min/1.73m2)** |  |  |
| >90 | reference |  |
| 60-90 | 1.11 [0.80;1.54] | 0.545 |
| 30-60 | 1.65 [0.96;2.82] | 0.070 |
| 15-30 | 1.81 [0.64;5.10] | 0.265 |
| <15 | 1.40[0.26;7.56] | 0.694 |
| **KDIGO stage** |  |  |
| Low risk | reference |  |
| Moderate risk | 1.52 [1.05;2.22] | 0.028 |
| High risk | 1.91 [1.05;3.49] | 0.035 |
| Very high risk | 2.14 [1.17;3.92] | 0.014 |

d Adjusted for gender, age, hypertension, diabetics, LDL, TG, AHF, CHF and CADRADSscore.
